# The Effects of Different Storage Temperatures on Chemical Substance Changes in Saffron Stigmas Analyzed by LC-MS and GC-TOF-MS

**DOI:** 10.3390/foods15203758

**Published:** 2026-10-21

**Authors:** Lin Zhou, Jiao Zhu, Xin Li, Linmei Shao, Zijin Xu, Luting Xing, Liuyan Yang, Yongchun Zhang

**Affiliations:** 1 Forest and Pomology Research Institute, Shanghai Academy of Agricultural Sciences, Shanghai 201106, China; zhoulin6816@126.com (L.Z.); zhujiao@saas.sh.cn (J.Z.); luguzi@163.com (X.L.); lingmeishao@saas.sh.cn (L.S.); 2 Department of Pharmacy, Jiangxi Medical College, Shangrao 334000, China; cczyyxzj@163.com; 3 Shanghai Agricultural Science and Technology Service Center, Shanghai 200335, China; xingluting8888@126.com

**Keywords:** saffron, stigmas, storage, non-targeted metabolism, chemical substances

## Abstract

This study evaluated the effects of three storage temperatures (4 °C, −20 °C, −80 °C) on metabolites in saffron stigmas after one year of storage using non-targeted metabolomics based on LC-MS and GC-TOF-MS. Lower storage temperatures better preserved chemical activity, and different metabolites markedly included capsiate, sarmentine, sesamolinol, nobiletin, prostaglandin F1α, vanillin, and α-ketoglutaric acid. Amino acid-centered pathways, including alanine, aspartate, glutamate, and phenylalanine metabolism, constitute core temperature-responsive modules. Aspartic acid and alpha-ketoisovaleric acid exhibited strong correlations with key quality indicators crocin and picrocrocin. The −80 °C treatment significantly yielded the highest retention of crocin and picrocrocin, with their contents being 21.46% and 31.25% higher, respectively, than at 4 °C. −80 °C is recommended for long-term storage, whereas −20 °C is suitable for household use. This study provides a theoretical foundation and practical guidance for saffron preservation for producers and consumers.

## 1. Introduction

Saffron (*Crocus sativus* L.) is an autumn herbaceous flowering plant of the iris family, which is mainly grown in Iran, India, and Morocco, and was introduced to China via the Silk Road in the Han Dynasty [1,2]. Saffron is one of the most expensive crops in the world of medicinal plants, and it is called ‘red gold’ [3]. The dried stigmas of saffron, as a time-honored medicinal herb, are rich in medicinal components such as crocin, safranal, picrocrocin, and other key secondary metabolites. Crocin is responsible for the color of the stigmas, whereas picrocrocin and safranal are responsible for its bitter taste and aroma [4,5]. It possesses a wide medicinal function of cardioprotective, neuroprotective, memory-enhancing, and prevention of dementia in patients, as well as antidepressant and anxiolytic effects [6,7,8,9,10]. In recent years, saffron has been studied for its application in a variety of diseases, including neuronal, cardiovascular and other systems as well as cancer [11,12,13,14].

Accumulating evidence has further validated a wide range of biological properties of saffron-derived compounds, covering anticancer, antibacterial, cosmetic, anti-inflammatory, antinociceptive, hypnotic, anxiolytic, anesthetic, antidepressant, and bronchodilatory activities [2]. In view of these beneficial properties, it is a common practice in China to brew 5–10 saffron stigmas in tea and alcoholic beverages for health preservation [2]. Stigma is the primary medicinal organ of saffron and requires long-term storage for future daily utilization after being dried. However, it had not been clear how different storage temperatures affect the medicinal substance activity of saffron stigmas. Temperature is the main environmental factor regulating plant secondary metabolites, especially flavonoids and terpenoids [15]. When environmental temperature decreased to 4–10 °C, the low-temperature stress would stimulate the accumulation of flavonoids and terpenoids [16]. Yoo found that quercetin glucoside content in onions increased by approximately 50% when it was stored at 30 °C environmental temperature for five months [17]. However, there was no significant change in quercetin content when onion bulbs were stored at 4 °C. Kałużewicz investigated the impact of temperature in short-time storage (6 and 24 h) at room temperature and cold storage for one week on the contents of quercetin, kaempferol, and vitamin C in broccoli heads [18]. They found that an increase in storage room temperature leads to an elevation in the contents of quercetin and kaempferol, accompanied by greater losses of vitamin C. A decrease in temperature for broccoli in storage may also result in a reduction in flavonoid content. Misai Kucing was stored at 5 °C, 10 °C, and 25 °C (room temperature) for four weeks, and it was found that the total phenolic, total flavonoid, and rosmarinic acid contents of fresh Misai Kucing significantly decreased during both low-temperature and ambient-temperature storage [19]. Daily storage typically employs temperatures of 4 °C and −20 °C, which help maintain better food stability under low-temperature conditions [20]. However, there is no systematic research clarifying the effects of storage temperature on saffron stigma’s chemical composition.

Therefore, we need to understand what the trends are for medicinal metabolites of saffron stigmas when stored at different environmental temperatures. Metabolomics is the global analysis of a large number of metabolites [21]. Non-targeted metabolomics, as an emerging technology in metabolomic research, enables precise and efficient qualitative and quantitative analysis of differential metabolites in biological systems. This technique has been widely applied across diverse fields, including food science, floriculture, crop research, and pomology, playing a pivotal role in the in-depth exploration of volatile flavor characteristics and compositional profiles [22,23,24,25]. Thereby, our study reported the effect of different storage temperatures on the changes in medicinal components of saffron stigmas using non-targeted metabolomics coupled with LC-MS and GC-TOF-MS technologies. We identified differential metabolites present in stigmas when stored at different environmental temperatures and elucidated the regulated medicinal metabolites of saffron stigmas. Our research aims to explore the optimal storage temperature for saffron stigmas, ensuring that important medicinal metabolites in stigmas remain unaffected in storage. Meanwhile, this study proposed providing technical guidance and a theoretical foundation for saffron stigmas storage and the application of saffron stigmas stored for consumers and producers.

## 2. Materials and Methods

### 2.1. Experimental Materials and Experiment Design

The materials of saffron stigmas in this experiment were selected from the Baihe Base of the Shanghai Academy of Agricultural Sciences. When fresh saffron stigmas were harvested from saffron flowers and dried immediately in a 60 °C drying oven to constant weight for long-term storage [26]. All saffron stigma materials in this study used for storage experiments were derived from the same batch of dried stigmas, with the purpose of avoiding differences among samples prior to storage. Subsequently, dried stigmas were stored separately in freezers at 4 °C (T1), −20 °C (T2), and −80 °C (T3) (DW-86L338J, Qingdao Haier Special Appliances Co., Ltd., Qingdao, Shandong province, China) for one year, and there were six independent dried stigmas samples in each different temperature storage. In detail, these dried stigmas in this study were kept in light-protected and sealed conditions during the storage period.

When the samples of dried stigmas reach a low water activity, not only is enzymatic metabolic activity within cells significantly inhibited, but the rates of most non-enzymatic chemical reactions (such as oxidation and Maillard reaction) are also substantially reduced. However, after long-term storage, it also would cause substrates and enzymes to interact, enabling residual enzymatic conversions that may occur during storage; as enzymes gradually inactivate, non-enzymatic chemical reactions such as oxidation, hydrolysis, and isomerization continue to drive compositional changes. Concurrently, some bound-state metabolites are progressively released, ultimately resulting in a large number of differential metabolites [26,27]. Therefore, after one year of storage, the stigmas in T1, T2, and T3, which had six independent biological replications for each treatment, were analyzed using non-targeted metabolomics methods with LC-MS and GC-TOF-MS technologies.

### 2.2. Experimental Methods

In this study, saffron stigmas were stored under three different temperature conditions and analyzed using non-targeted metabolomics coupled with LC-MS and GC-TOF-MS technologies in DDA mode after one year of storage.

#### 2.2.1. Liquid Chromatography–Tandem Mass Spectrometry (LC-MS) Analysis

(1) Sample Preparation: Saffron stigmas were weighed 1 g for each replication and were freeze-dried under vacuum and then ground into a powder using a ball mill (35 Hz, 4 min). A total of 20 mg of the sample powder was accurately weighed and dissolved in 1000 μL of pre-cooled extraction solution (methanol:water = 3:1 (*v*/*v*), containing isotope-labeled internal standard mixture). To ensure complete dissolution, the sample was sonicated three times (5 min in an ice-water bath), and then the mixture was left standing at −40 °C for 1 h. Afterward, it was centrifuged at 4 °C for 15 min, and the supernatant was collected. The sample was filtered using a 0.22 μm micropore filter and stored in an injection vial for LC-MS analysis.

(2) Chromatographic and Mass Spectrometry Analysis: LC-MS analysis employed a Vanquish (Thermo Fisher Scientific, Shanghai, China) ultra-high-performance liquid chromatograph for the chromatographic separation of target compounds using a Waters ACQUITY UPLC HSS T3 chromatographic column (2.1 mm × 100 mm, 1.8 μm). Mobile phase A consisted of an aqueous solution containing 5 mmol/L ammonium acetate and 5 mmol/L acetic acid, while mobile phase B was acetonitrile. The sample tray temperature was set to 4 °C, and the injection volume was 2 μL. The chromatographic conditions were as follows: chromatographic column, Waters ACQUITY UPLC HSS T3 (2.1 mm × 100 mm, 1.8 μm); mobile phase A, 5 mmol/L ammonium acetate and 5 mmol/L acetic acid; mobile phase B, acetonitrile; gradient elution program: 0–0.7 min, 1% B, flow rate 0.35 mL/min; 0.7–9.5 min, 1–99% B, flow rate 0.35 mL/min; 9.5–11.8 min, 99% B, flow rate 0.35–0.5 mL/min; 11.8–12 min, 99–1% B, flow rate 0.5 mL/min; 12–14.6 min, 1% B, flow rate 0.5 mL/min; 14.6–14.8 min, 1% B, flow rate 0.5–0.35 mL/min; 14.8–15 min, 1% B, flow rate 0.35 mL/min; column temperature 35 °C, injection port temperature 4 °C. The Orbitrap Exploris 120 mass spectrometer was controlled by the Xcalibur software (version: 4.4, Thermo) for the acquisition of both primary and secondary mass spectral data.

(3) Data Analysis: After converting the raw data into mzXML format using ProteoWizard software (Version 3.0), peak identification, extraction, alignment, and integration were performed using a self-developed R package (with XCMS as its core). Subsequently, metabolite annotation was conducted by matching against a self-built secondary mass spectrometry database (BiotreeDB V2.1), with a cutoff value of 0.3 for algorithm scoring. A score of 0.3 was set as the secondary mass spectrometry matching score threshold, where a higher score indicates a better match with database spectra. Metabolite identification in the samples was carried out by matching against information such as retention times, parent ion mass-to-charge ratios (with molecular mass errors within <10 ppm), and secondary fragmentation spectra of metabolites in the self-built BiotreeDB (V2.1) secondary mass spectrometry database.

The identification results were annotated according to the levels of metabolite identification described in Alseekh’s literature [28]: Level A: Standard compound-targeted validation or NMR identification. Level B(i): Reliable MS/MS matching based on the standard compound database. Level B(ii): Reliable MS/MS matching based on the simulated database; Level B(iii): Partial MS/MS matching. Level C(i): Reliable MSn matching based on the standard compound database. Level C(ii): Reliable MSn matching based on the simulated database. Level C(iii): Partial MSn matching; Level D: MS-based matching.

#### 2.2.2. Gas Chromatography–Time-of-Flight Mass Spectrometry (GC-TOF-MS) Analysis

(1) Sample Preparation: The saffron stigmas were freeze-dried under vacuum and ground into powder using a ball mill (40 Hz, 4 min). A total of 20 ± 1 mg of the sample powder was accurately weighed and placed in a 2 mL EP tube. A total of 1000 μL of pre-cooled extraction solution (methanol:water = 3:1 (*v*/*v*), containing internal standard ribitol) was added and vortexed for 30 s. To ensure complete dissolution, steel beads were added to the EP tube, and the samples were sonicated in an ice-water bath for 5 min (repeated three times). After sonication, they were centrifuged at 4 °C for 15 min, and 50 μL of the supernatant was taken into a 1.5 mL EP tube. A total of 50 μL of each sample was combined to prepare the QC sample. The extract was then dried in a vacuum concentrator, and 60 μL of methoxyamine hydrochloride reagent (dissolved in pyridine at 20 mg/mL) was added to the dried metabolites. After mixing, the samples were incubated at 80 °C for 30 min. Next, 80 μL of BSTFA (containing 1% TMCS, *v*/*v*) was added, and the mixture was incubated at 70 °C for 1.5 h. After the samples cooled to room temperature, 5 μL of FAMEs (dissolved in chloroform) was added. Finally, the samples were stored in injection vials for GC-TOF-MS analysis.

(2) Chromatographic and Mass Spectrometry Analysis: This was performed by adopting the experimental method of Wiklund [29]. GC-TOF-MS analysis was performed using an Agilent 7890 gas chromatograph (Agilent Technologies, Inc., Santa Clara, CA, USA) coupled with a time-of-flight mass spectrometer. The system utilized a DB-5MS capillary column, with 1 μL of sample injected in splitless mode. Helium was used as the carrier gas, with a front inlet purge flow of 3 mL/min and a column flow rate of 1 mL/min. The initial temperature was set to 50 °C and held for 1 min, then increased at a rate of 10 °C/min to 310 °C, where it was held for 8 min. The injection port, transfer line, and ion source temperatures were set to 280 °C, 280 °C, and 250 °C, respectively. In electron impact mode, the energy was set to −70 eV. Mass spectrometry data were acquired in full scan mode from 50 to 500 m/z at a rate of 12.5 spectra per second after a solvent delay of 6.4 min.

(3) Data Analysis: The mass spectrometry data were analyzed using ChromaTOF software (V 4.3x, LECO, St. Joseph, MI, USA) for peak extraction, baseline correction, deconvolution, peak integration, and peak alignment. In the qualitative analysis, the LECO-Fiehn Rtx5 database was used for spectral matching and retention time index matching. Low-quality peaks were eliminated to ensure data reliability: peaks present in fewer than 50% of quality control (QC) samples or with a relative standard deviation (RSD) greater than 30% across QC samples were removed for subsequent analysis.

All annotated metabolites were revalidated and assigned transparent confidence levels according to the metabolite annotation framework reported by Alseekh [28], which is universally applicable to GC-TOF-MS metabolomics data. Metabolite identification was performed based on accurate mass, retention index, and EI full fragmentation spectral matching. The confidence levels were defined as follows: Level A, verification by authentic chemical standards or NMR identification; Level B(i), reliable matching with standard database EI fragmentation spectra; Level B(ii), reliable matching with in silico database EI fragmentation spectra; Level B(iii), partial matching of EI fragmentation spectra; Level D, preliminary identification based on primary mass spectral matching.

### 2.3. Determination of Mass Concentration Percentage of Crocin and Picrocrocin at Different Storage Temperatures

We determined the mass concentration percentage of crocin and picrocrocin in saffron stigmas after drying immediately, which is recorded as T0 without storage. Subsequently, dried stigmas of T0 were stored for one year under different storage temperatures (4 °C (T1), −20 °C (T2), and −80 °C (T3)), after which their mass concentration percentage of crocin and picrocrocin content was determined. All saffron stigma materials used for storage experiments were derived from the same batch of dried stigmas and subjected to identical drying procedures at the same time.

Quantification of crocin and picrocrocin content was carried out on pooled samples from each of two independent biological groups (*n* = 3). This experiment for determination was applied to high-performance liquid chromatography with the single-point external standard method. In detail, the mass concentration percentages of crocin and picrocrocin were determined in accordance with their methods in the *Chinese Pharmacopoeia* (2020 edition), and the specific operational steps for the mass concentration percentage of crocin and picrocrocin can be referred to in Zhou’s method [30] with three replications for each indicator.

The dried saffron stigmas were accurately weighed, homogenized by mechanical grinding, and sieved to obtain fine and uniform powder. Subsequently, 10 mg of the prepared stigma powder was added to dilute ethanol for solvent extraction. Ultrasonic-assisted extraction was carried out in an ice bath for 20 min under fixed ultrasonic parameters of 50 kHz and 300 W. After extraction, the resulting solution was diluted with dilute ethanol to a final volume of 50 mL and filtered to acquire the clarified test solution for subsequent chromatographic detection. All HPLC measurements were conducted at a constant temperature of 25 °C using a Diamonsil C18 column (250 × 4.6 mm, 5 μm). The mobile phase system consisted of acetonitrile (phase A) and ultrapure water (phase B), with a gradient elution program configured as follows: 13% phase A from 0 to 20 min, 23% phase A from 20 to 23 min, 25% phase A from 23 to 45 min, and 50% phase A from 45 to 50 min. The mobile phase flow rate was set steadily at 1.0 mL/min, and the sample injection volume was 10 μL. The maximum absorption wavelengths were determined as 440 nm for crocin (crocin I and crocin II) and 254 nm for picrocrocin, which were adopted as the detection wavelengths for the two target compounds. The calculation formulas for the mass concentration percentages of crocin and picrocrocin are displayed below.

The mass concentration percentages of crocin in saffron samples were quantified using the following calculation equation: CC = CC_R_ × A_X_/A_R_ × 100%. In this formula, CC refers to the mass concentration percentage of crocin in experimental samples; CC_R_ corresponds to the mass concentration percentage of the crocin reference standard, with individual purities of crocin I and crocin II being 90.7% and 89.2%, respectively. All reference standards were sourced from Shanghai Standard Technology Co., Ltd., Shanghai, China. A_X_ indicates the chromatographic peak area of the tested sample recorded at 440 nm, whereas A_R_ represents the peak area of the standard substance detected under the same wavelength condition. The relative standard deviation (RSD) of the peak area was 0.262% for crocin I and 0.633% for crocin II. Notably, the total crocin content of the standard was defined as the combined content of crocin I and crocin II.

Meanwhile, the quantitative calculation of mass concentration percentages of picrocrocin was conducted using the formula PC = PC_R_ × A_X_/A_R_ × 100%. Here, PC denotes the mass percentage of picrocrocin in experimental samples, while PC_R_ stands for the mass percentage of picrocrocin in the reference standard, whose purity percentage was 97.30% (Shanghai Standard Technology Co., Ltd., China). A_X_ and A_R_ separately represent the peak areas of the sample and standard determined at the detection wavelength of 254 nm. The RSD value of the picrocrocin peak area in this detection method was calculated as 0.184%.

### 2.4. Statistical Analysis

All experimental data were systematically sorted and compiled in Microsoft Excel 2007. Statistical evaluations, including differential significance analysis, variance analysis, and correlation analysis, were performed using IBM SPSS Statistics 23.0 for Windows. Bar graph visualization of quantitative data was done with Origin 8.0 software from OriginLab Corporation. For multivariate statistical analysis and bioinformatics visualization, professional tools were used according to analytical requirements: principal component analysis (PCA) with SIMCA (version 16.0.2); Venn diagrams and upset plots via the VennDiagram (version 1.6.20) and UpSetR (version 1.4.0) R packages (version 3.3.5); volcano plots and KEGG functional enrichment analysis on the R (version 3.3.5) platform; and correlation heatmaps using the R corrplot package (version 0.89).

## 3. Results and Discussion

### 3.1. Principal Component Analysis (PCA), Metabolite Classification, and Volcano Plot Analysis of Differential Metabolites in T1, T2, and T3

**Figure 1:**
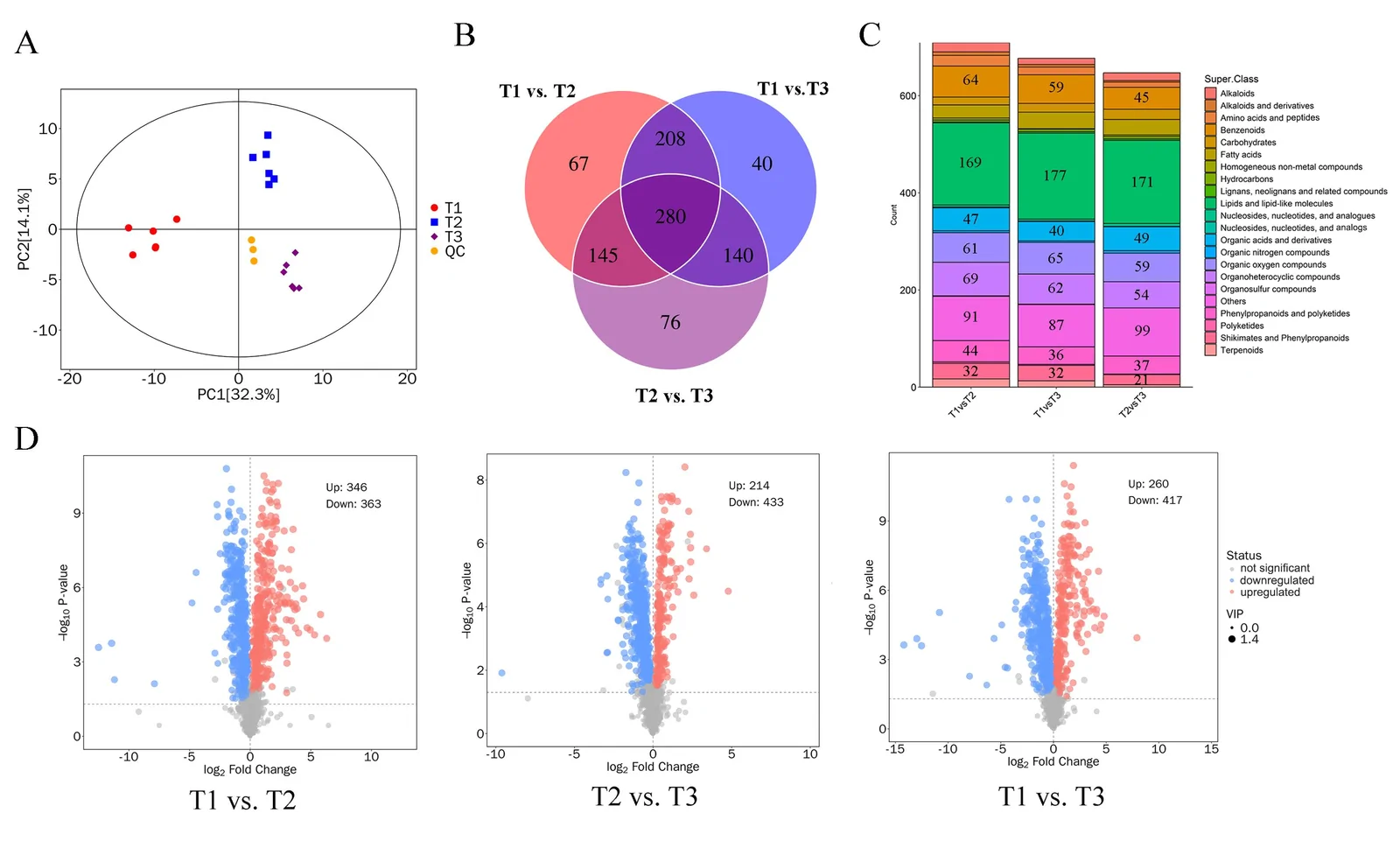
(**A**) PCA; (**B**) Venn; (**C**) metabolite classification; (**D**) volcano plot analysis of differential metabolites in T1, T2, and T3. Note: The threshold for screening differential metabolites between groups was set at VIP > 1 and *p* < 0.05. The Venn diagram counts the total number of compounds that show significant differences in the corresponding comparisons, and it performs statistical analysis based on substances with compound names (unique metabolites after deduplication). QC sample: all samples of T1, T2, and T3, which total 18 replications with mixing in equal volumes. The up- and downregulated counts in volcano plots refer to the total number of peaks that meet the criteria of VIP > 1 and *p* < 0.05, and the volcano plot was generated based on the statistical analysis of peak ID counts.

PCA was conducted on the overall metabolites of three treatment samples (T1, T2, and T3) to gain a preliminary understanding of the overall metabolite differences between groups and variability within each group. The PCA results show a trend of separation in overall metabolites among the three groups, with a tendency for samples within the same group to cluster together. As seen in Figure 1A, main components PC1 and PC2 contribute 32.3% and 14.1% to the differences, respectively. This indicates that there are significant differences in overall metabolites of saffron stigma samples stored at different temperatures. The overall metabolite data detected in stigma samples were summarized and analyzed, and the three samples were grouped (T1 vs. T2, T2 vs. T3, and T1 vs. T2) for comparison. An Orthogonal Partial Least Squares Discriminant Analysis (OPLS-DA) model was established for analysis (Figure S1). The closer the Q^2^ and R^2^Y values of the OPLS-DA model are to 1, the more stable and reliable the model is. The OPLS-DA models for the T1 vs. T2, T1 vs. T3, and T2 vs. T3 comparison groups were subjected to 200 permutation tests. The results show that the Q^2^ and R^2^Y values are 0.972 and 0.978, 0.998 and 0.997, and 0.926 and 0.995, respectively. The *p*-values were all less than 0.05, indicating that there are significant differences in the types and quantities of overall metabolites present in the three saffron stigma samples.

Through comparative analysis of the metabolome of saffron stigmas stored at different temperatures, a total of 956 metabolites were identified (Figure 1B). Specifically, three groups of T1 vs. T2, T2 vs. T3, and T1 vs. T3 contained 700, 641, and 668 metabolic components, respectively, with 280 common metabolites shared among the three. Among them, there were 67 unique metabolites in T1 vs. T2, 76 unique metabolites in T2 vs. T3, and 40 unique metabolites in T1 vs. T3. To further understand which classes these differentially expressed metabolites belong to, we conducted an analysis of metabolite classification and quantity in Figure 1C. Among these metabolic components, lipids and lipid-like molecules (29.24%), organic heterocyclic compounds (12.79%), organic oxygen compounds (12.72%), and organic acids and their derivatives (12.35%) were abundant. It is worth noting that the number of differential metabolites was the smallest in T2 vs. T3, while the number of differential metabolites in T1 vs. T2 was the largest. In detail, there was the largest number of differential metabolites in lipids and lipid-like molecules, and their numbers were 169, 177, and 171, respectively, in T1 vs. T2, T2 vs. T3, and T1 vs. T3. Our results indicate that there are significant differences in metabolite kinds and numbers in saffron stigmas when stored in different temperature environments, especially in the lipids and lipid-like molecules class.

To understand the differences in composition of differential metabolites among saffron stigmas in three treatments, the *t*-test (*p* < 0.05) and VIP value (VIP > 1) from OPLS-DA were used for screening between each group. As shown in the volcano plot of differential metabolites (Figure 1D), in T1 vs. T2, 346 metabolites had increased relative content and 363 had decreased relative content of stigmas in T1 compared with T2. In T2 vs. T3, 641 differential metabolites were identified, with 214 metabolites showing increased relative content and 433 decreased relative content in T2 compared with T3. In T1 vs. T3, 260 metabolites relatively were upregulated, and 417 metabolites were downregulated in T1 compared with T3.

### 3.2. Screening and Analysis of Top 15 Differential Metabolites

**Figure 2:**
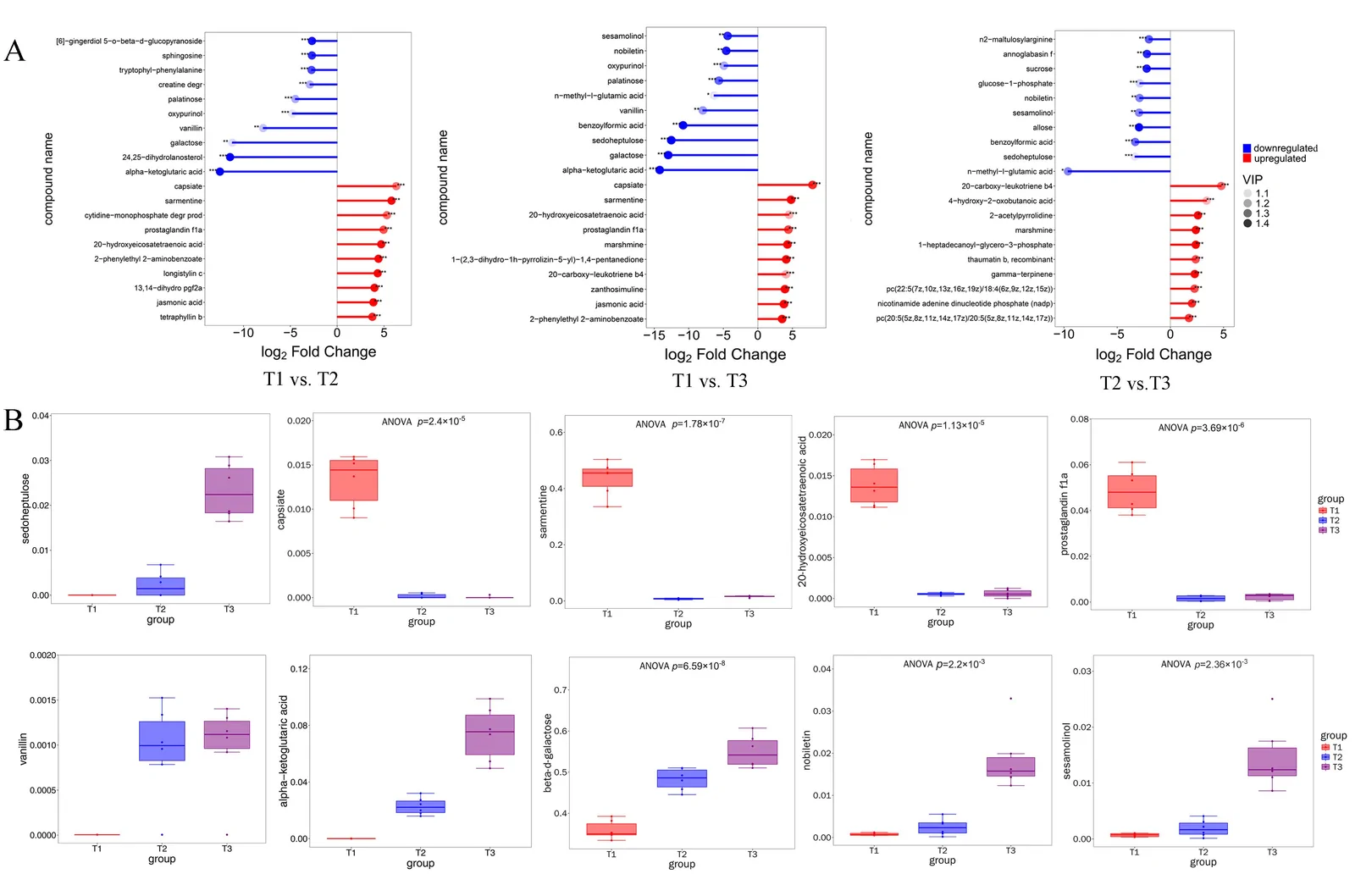
(**A**) matchstick plots of differential metabolites in T1 vs. T2, T1 vs. T3, and T2 vs. T3; (**B**) boxplots of relative abundance of important metabolites in T1, T2, and T3. Note: The threshold for screening differential metabolites between groups was set at VIP > 1 and *p* < 0.05. ANOVA Model: One-way analysis of variance (one-way ANOVA) was employed, with the factor being ‘storage temperature’ (T1, T2, T3) and the dependent variable being the peak areas of each metabolite (after log transformation and z-score standardization). * indicated there was significantly difference at *p* < 0.05; ** indicated there was significantly difference at *p* < 0.01; *** indicated there was significantly difference at *p* < 0.001. The ANOVA *p*-values are presented in scientific notation, and data are presented as mean ± SD (*n* = 6).

Previous studies reported saffron stigmas possess multiple pharmacological activities, including anticancer, antioxidant, antibacterial, antifungal, and anti-inflammatory effects, as well as the functions of alleviating depression and improving memory [31]. The top 15 metabolites with the highest fold changes in upregulation and downregulation were selected for result presentation (Figure 2A,B). In T1 vs. T2, metabolites with decreased relative abundance included α-ketoglutaric acid, 24,25-dihydrolanosterol, galactose, vanillin, and oxypurinol, among others, while those with increased relative abundance included capsiate, sarmentine, cytidine-monophosphate degr prod, prostaglandin F1α, and 20-hydroxy-eicosatetraenoic acid, among others. In T2 vs. T3, the metabolites with increased relative abundance included 20-carboxyleukotriene B4 and 4-hydroxy-2-oxobutanoic acid, 2-acetylpyrrolidine, and marshmine, among others, while those with decreased relative content included N-methyl-L-glutamic acid, sedoheptulose, and benzoylformic acid, among others. In T1 vs. T3, differential metabolites with increased relative abundance included capsiate, sarmentine, 20-hydroxyeicosatetraenoic acid, prostaglandin F1α, and marshmine, among others, while those with decreased relative abundance included α-ketoglutaric acid, galactose, sedoheptulose, benzoylformic acid, and vanillin, among others.

Compared with T3, both T1 and T2 showed increased marshmine, accompanied by decreased sedoheptulose and benzoylformic acid. The decreased sedoheptulose suggested carbon-flow redistribution in the pentose-phosphate pathway, with carbon skeletons preferentially channelled toward defensive secondary metabolism rather than accumulating as free heptose sugar [32]. Several metabolites, including capsiate, sarmentine, prostaglandin F1α and 20-hydroxyeicosatetraenoic acid, were consistently elevated in T1 relative to both T2 and T3, while α-ketoglutaric acid, galactose and vanillin were consistently reduced in T1. Relative abundance of metabolites, including capsiate, sarmentine, prostaglandin F1α and 20-hydroxyeicosatetraenoic acid, was significantly enhanced in saffron stigmas under T1 compared with T2 and T3. Capsiate and sarmentine are known to exhibit antioxidant and antimicrobial properties, presumably synergistically suppressing pathogen proliferation and reducing mildew and spoilage during stigma storage [33,34]. They may also scavenge reactive oxygen species, alleviate lipid peroxidation, delay postharvest senescence, and protect characteristic bioactive components such as crocin. The 20-hydroxyeicosatetraenoic acid and prostaglandin F1α belong to oxylipin-type lipid mediators, which are induced upon oxidative stress and tissue injury, participating in lipid remodeling and stress signal transduction in plants [35]. The co-increase of these metabolites suggests that T1 is associated with secondary defense and oxylipin-dependent stress-responsive networks and improves the storage performance of saffron stigmas.

This study indicates that the relative abundance of vanillin and α-ketoglutaric acid was significantly decreased in the T1 treatment compared with T2 and T3. It is worthy to notice that vanillin is a known natural aromatic flavoring agent and plays a crucial role in the pharmacological activities of vanillin, including anticancer, antidiabetic, antioxidant, anti-sickling, antimicrobial, anti-inflammatory, aphrodisiac, cardioprotective, and diuretic actions [36]. Meanwhile, we further speculated that saffron stigmas stored at 4 °C would cause the fragrance to fade and cause a reduction in critical medicinal substance components compared with other temperatures. On the other hand, α-ketoglutaric acid could significantly reduce browning of peels and leaves, delay pigment degradation, and preserve cell membrane integrity [37,38]. Our results indicate that saffron stigmas in T1 would easily decline the medicinal quality of T1 compared with T2 and T3, which is not a good storage temperature for stigmas.

In this study, we found that there were the largest fold changes in downregulation in T1 vs. T3; conversely, the largest fold changes in upregulation were shown in T1 vs. T2. It indicated that there were lots of differential metabolites in T1, and their contents significantly decreased compared with T2 and T3. Meanwhile, it indicated that there were greater differences in the relative abundance of important medicinal metabolites for saffron stigmas when storage temperature had greater differences. In detail, the relative abundance of these metabolites, such as alpha-ketoglutaric acid, galactose, sedoheptulose, and benzoylformic acid, was significantly decreased in T1 compared with T3. On the other hand, the relative abundance of alpha-ketoglutaric acid, 24,25-dihydrolanosterol, galactose, and vanillin was significantly downregulated in T1 compared with T2. It indicated that two metabolites of alpha-ketoglutaric acid and galactose were significantly decreased in T1 compared with T2 and T3.

On the other hand, we found that saffron stigmas that were stored in a higher-temperature environment (T1) for storing stigmas would decrease the relative abundance of chemical substances such as nobiletin, sedoheptulose, benzoylformic acid, n-methyl-l-glutamic acid, and sesamolinol compared with T2 and T3. In detail, nobiletin is a naturally occurring compound that has wide-ranging pharmacological activities, including neuroprotection, cardiovascular protection, antimetabolic disorder, anticancer, anti-inflammation, and antioxidation [39]. Sesamolinol and its glucosides are recognized for their potent antioxidant activities, and they also function in reducing inflammation, lowering blood lipid levels, and reducing blood pressure [40]. Conversely, marshmine and 20-carboxy-leukotriene had higher relative abundances in T1 and T2 compared with T3. Previous studies reported these two chemical substances had anti-inflammatory and antibacterial properties [41]. Our results indicate that storage temperature would affect the content of effective medicinal components of saffron stigmas, and a lower-temperature environment is more suitable for stigmas storage to ensure medicinal efficacy.

### 3.3. K-Means Clustering and KEGG Analysis of Differential Metabolites in Different Treatments

**Figure 3:**
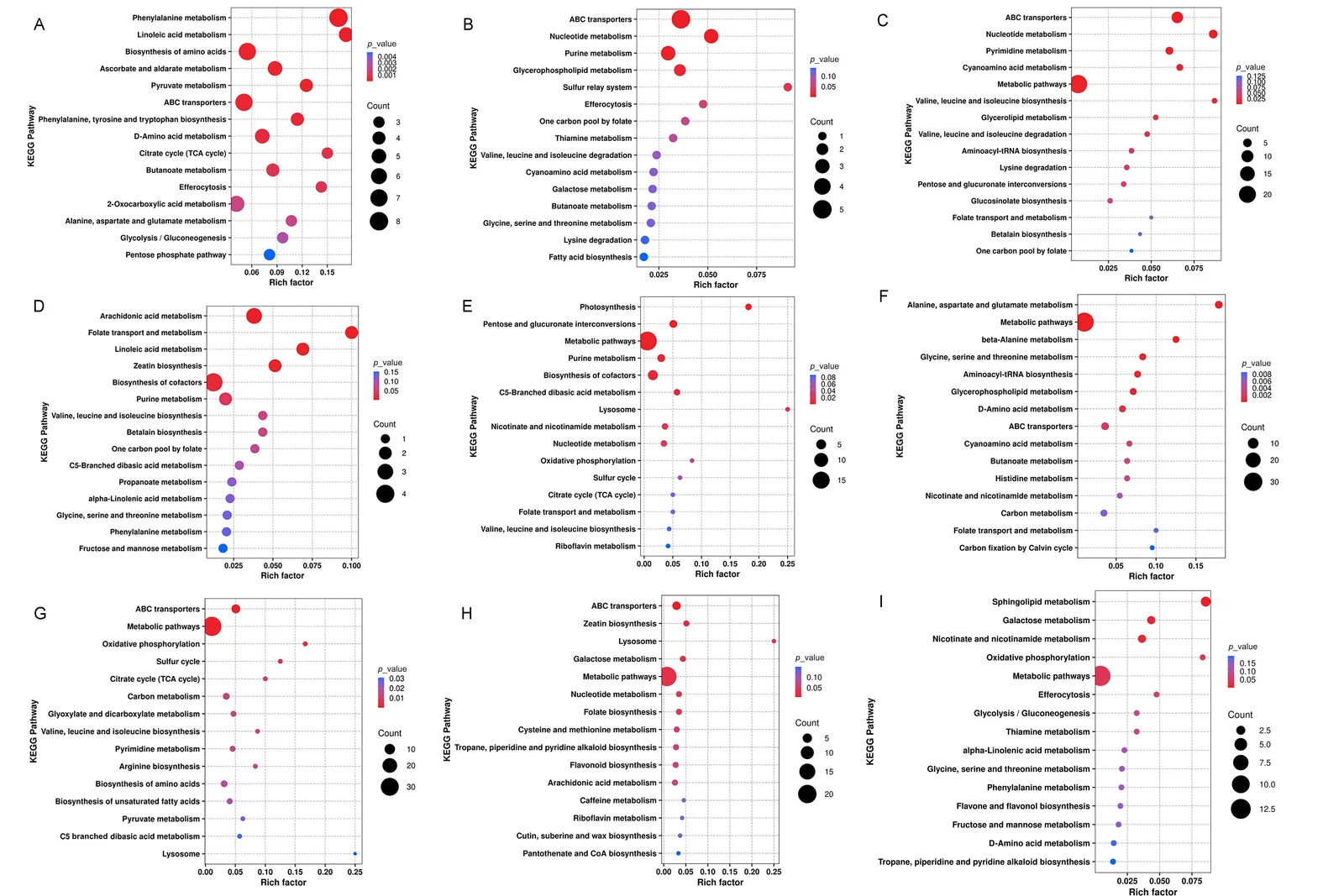
KEGG analysis of differential metabolites in K-means analysis: (**A**) KEGG analysis of Cluster 1; (**B**) KEGG analysis of Cluster 2; (**C**) KEGG analysis of Cluster 3; (**D**) KEGG analysis of Cluster 4; (**E**) KEGG analysis of Cluster 5; (**F**) KEGG analysis of Cluster 6; (**G**) KEGG analysis of Cluster 7; (**H**) KEGG analysis of Cluster 8; (**I**) KEGG analysis of Cluster 9. Note: Clustering Method: Metabolites were clustered using k-means hierarchical clustering on z-score standardized metabolites; the number of clusters k was set to 9 by default. KEGG pathway enrichment analysis: KEGG [R (version 3.3.5)] was utilized; the organism mapping was set to the corresponding species, *Dendrobium catenatum*; the background metabolite set was all metabolites detected in this study. Enrichment Test: The hypergeometric test was employed, and the *p*-values indicating enrichment significance were reported in the overall enrichment analysis table.

To investigate the trends in the relative abundance of metabolites in different treatments, we performed z-score normalization on the relative abundances of all differential metabolites identified according to screening criteria and K-means clustering analysis (Figure S2). Meanwhile, KEGG pathway enrichment analysis for K-means revealed distinct functional characteristics for each metabolite cluster in Figure 3. The results show that metabolites with consistent trends across the three samples could be classified into nine clusters.

Cluster 1 and Cluster 6 had completely opposite trends for each other, with 174 and 156 differential metabolites, respectively. The former showed a continuous upregulation trend in T1, T2, and T3, and they were concentrated on phenylalanine metabolism and linoleic acid metabolism, with its core biological function centered on amino acid metabolism. Previous studies reported that they were significantly enriched in linoleic acid metabolism in oat responses to heat stress, a pathway enrichment involved in membrane lipid oxidation and antioxidant response [42]. The invasion of pathogens into plant cell walls is the primary trigger for activating the plant defense phenylpropanoid metabolic pathway [43]. During storage, phenylalanine metabolism is associated under stress conditions. It modulates the intracellular phenylalanine pool and supplies cinnamic acid precursors for phenylpropanoid biosynthesis, further promoting the accumulation of phenolic acids, flavonoids and lignin. This pathway participates in postharvest disease resistance, antioxidant response and wound repair, and shapes the dynamic changes of bioactive constituents in medicinal plants [43]. Our results indicate that a lower-temperature environment could stimulate more pathways that function in antioxidant response for medicinal plants; it is very important for the activity of medicinal substance components. On the other hand, Cluster 6 showed a continuous downregulation trend in three treatments, and they were significantly enriched in metabolic pathways associated with the glutamate amino acid family.

The metabolites in Cluster 2 and Cluster 4 remained stable in T1 and T2 treatments, with 63 and 50 differential metabolites, respectively. Subsequently, the metabolites in Cluster 2 showed a continuous upregulation trend in T3, while those in Cluster 4 gradually downregulated in T3. Cluster 2 exhibited prominent enrichment in nucleotide metabolism. On the other hand, Cluster 4 showed an obvious downregulation trend in T3, and they were significantly enriched in linoleic acid metabolism, whose signaling pathways respond strongly. Our results indicate that there were lots of differential metabolites in linoleic acid metabolism, and the relative abundance of these differential metabolites in linoleic acid metabolism showed differences between T3 and T1 and T2. Previous studies reported that the most predominant fatty acids in all samples were linoleic acid (C18:2), linolenic acid (C18:3), and palmitic acid (C16:0), and higher levels of these fatty acids were reported in the dry stigmas [44]. Different storage temperature environments influence the non-enzymatic oxidation process of linoleic acid in dried stigmas. Oxidation of linoleic acid generates peroxides and free radicals, which further synergistically drive the oxidative degradation of crocin, while simultaneously producing some volatile secondary products in T1 and T2 [45]. Together, these processes contribute to the loss of saffron pigments and flavor alteration. It is consistent with our results that there were lower crocin contents in T1 and T2 compared with T3.

Cluster 3 and Cluster 7 exhibited similar relative abundance patterns, containing 80 and 112 differential metabolites, respectively. Their metabolites showed a continuous downregulation trend in T1 and T2 treatments and gradual upregulation in T3 compared with T2. For Cluster 3, KEGG enrichment shown in nucleotide metabolism. Cluster 7 was characterized by enriched pathways involved in energy metabolism and metabolic changes of sulfur-containing compounds. For Cluster 5, which had an opposite trend compared with Cluster 3, their metabolites showed a continuous upregulation trend in T2 treatment and gradual downregulation in T3 compared with T2. KEGG enrichment results indicate the most remarkable enrichment in metabolic pathways associated with oxidative phosphorylation, which governs energy metabolism.

Meanwhile, Cluster 8 had a similarly increased trend with Cluster 1, and the difference was mainly cumulative changes in T2, followed by a little increase in T3 compared with T2. On the other hand, compared with Cluster 8 and Cluster 9, the difference was shown in T3, and there was a decreased trend in T3 compared with T2. Both Cluster 8 and Cluster 9 displayed major enrichment in flavonoid metabolism and energy metabolism pathways. It is worth noting that there was an increasing trend in flavonoid metabolism in Cluster 8. Previous studies reported that saffron’s dried stigma tissue contains relatively high levels of these flavonoids, which possess high antioxidant capacity and may serve as potential health-promoting components in saffron [46]. In this study, metabolites such as isoquercitrin, 2″-(6-acetylglucosyl)astragalin, quercetin, cyanidin 3-(4-acetylglucoside), azaleatin 3-rutinoside, 6″-o-(3-hydroxy-3-methylglutaroyl)astragalin, nobiletin, kaempferol 3-[2″-glucosyl-6″-acetyl-galactoside] 7-glucoside, paeonoside, biorobin, kaempferitrin, orientin, quercetin 7-glucuronide 3-rhamnoside, rubraflavone a, kaempferol 3-sophorotrioside 7-rhamnoside, myricetin 3,3′-digalactoside, quercetin 3,4′-diglucoside, isorhamnetin, isorhamnetin 3-beta-laminaribioside, and dihydrokaempferol had higher quantitative values in the T2 and T3 compared to T1. In summary, active components of saffron stigmas are affected by storage temperature, and higher-temperature conditions would decrease important metabolite contents for medicinal value, including flavonoids and linoleic acid.

### 3.4. Functional Annotation and Enrichment Analysis of Differential Metabolites Based on KEGG Analysis

**Figure 4:**
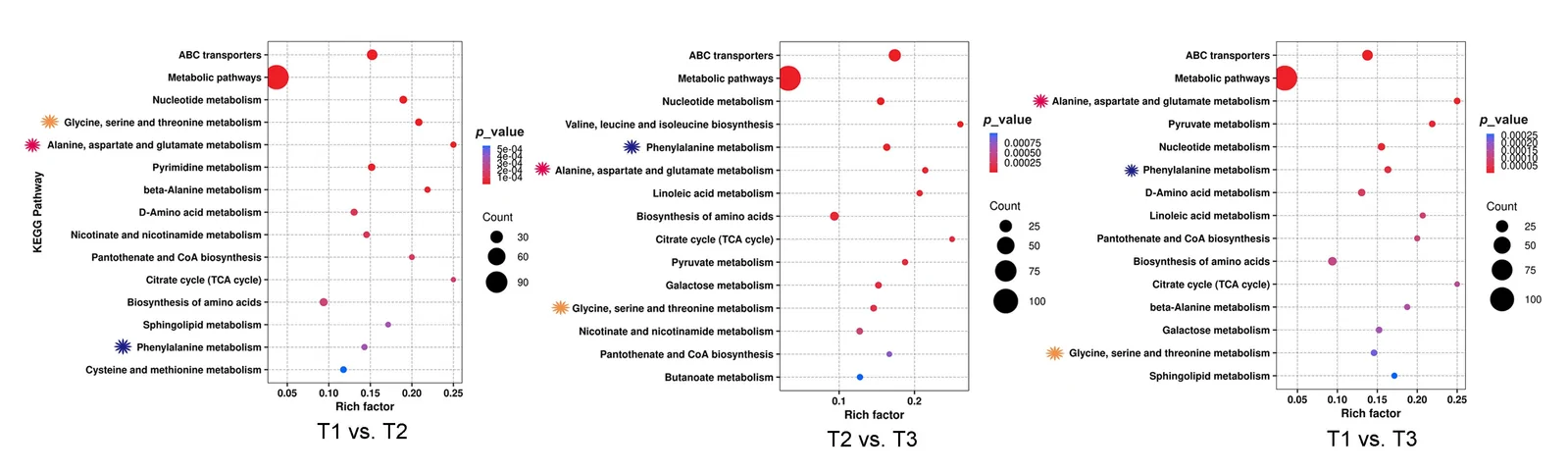
KEGG analysis of differential metabolites in T1 vs. T2, T2 vs. T3, and T1 vs. T3. Note: Clustering Method: Metabolites were clustered using k-means hierarchical clustering on z-score-standardized metabolites. KEGG pathway enrichment analysis was conducted by R software (version 3.3.5) for KEGG. The background metabolite set was all metabolites detected in this study. Enrichment Test: The hypergeometric test was employed, and the *p*-values indicating enrichment significance were reported in the overall enrichment analysis.

We conducted enrichment analysis on metabolic pathways involved by differential metabolites in different compared treatments (Figure 4). In T1 vs. T2, primarily characterized by changes related to amino acid, nucleotide, and substance transport; secondly, in T2 vs. T3, energy metabolism (tricarboxylic acid cycle) and lipid metabolism (linoleic acid) are further perturbed; finally, in T1 vs. T3, which functions on cumulative effect, amplifies overall differences, with the most significant effects observed in the alanine, aspartate, and glutamate metabolic pathways.

It is worth noting that there were five common differential metabolism pathways between them, such as biosynthesis of amino acids, TCA cycle, glycine, serine and threonine metabolism, alanine, aspartate and glutamate metabolism, and phenylalanine metabolism. Meanwhile, linoleic acid metabolism and galactose metabolism also play important roles in regulation in dried stigma storage. Amino acid metabolism is the most core responsive pathway throughout the different T1, T2, and T3 temperature environments. Among these, alanine, aspartate, and glutamate metabolism are significantly enriched in all three comparisons and are therefore the primary focus of attention, followed by glycine, serine, and threonine metabolism and phenylalanine metabolism.

### 3.5. Relative Differential Metabolite Abundance in Important Metabolism Pathways at Different Storage Temperatures for Saffron Stigmas

**Figure 5:**
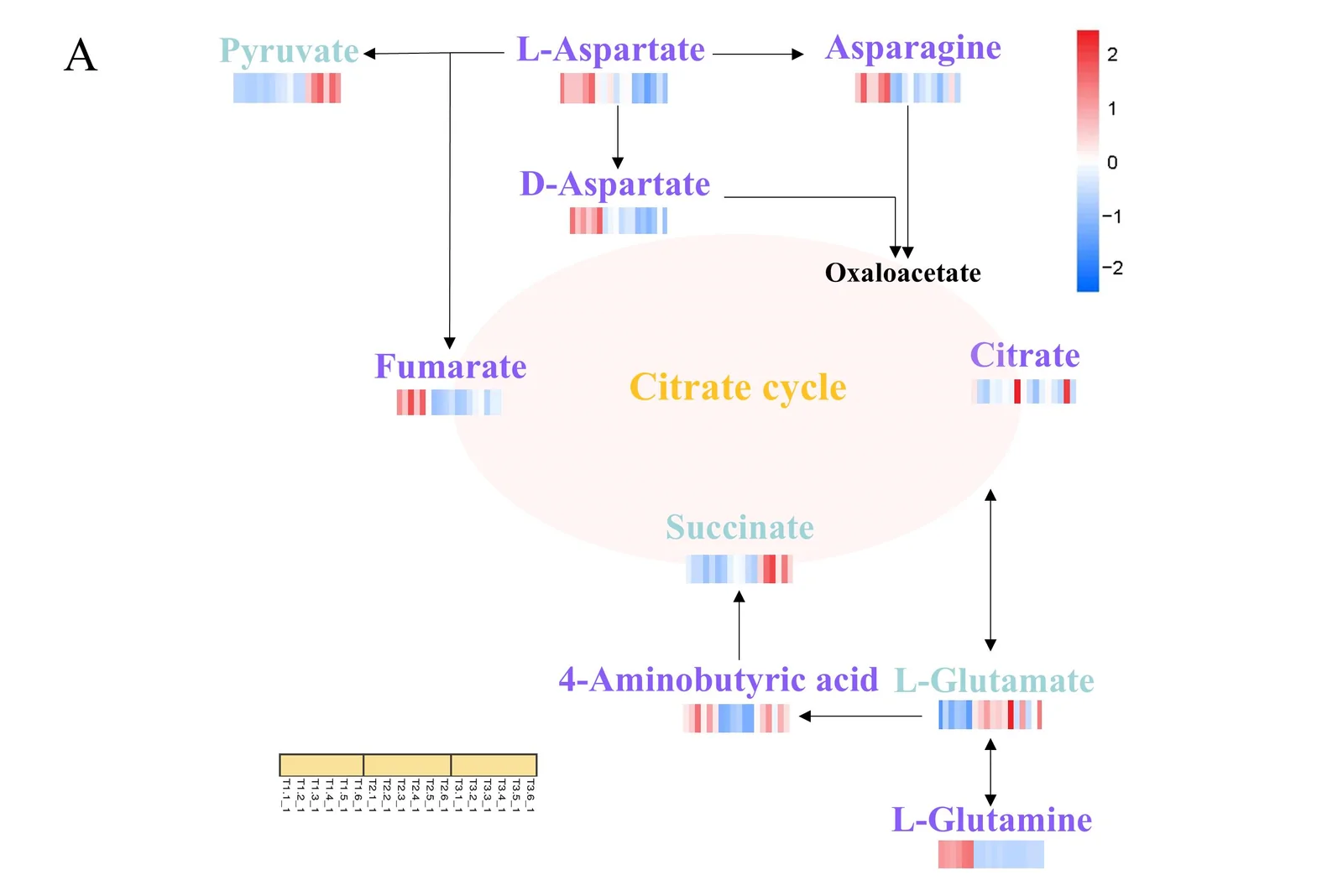

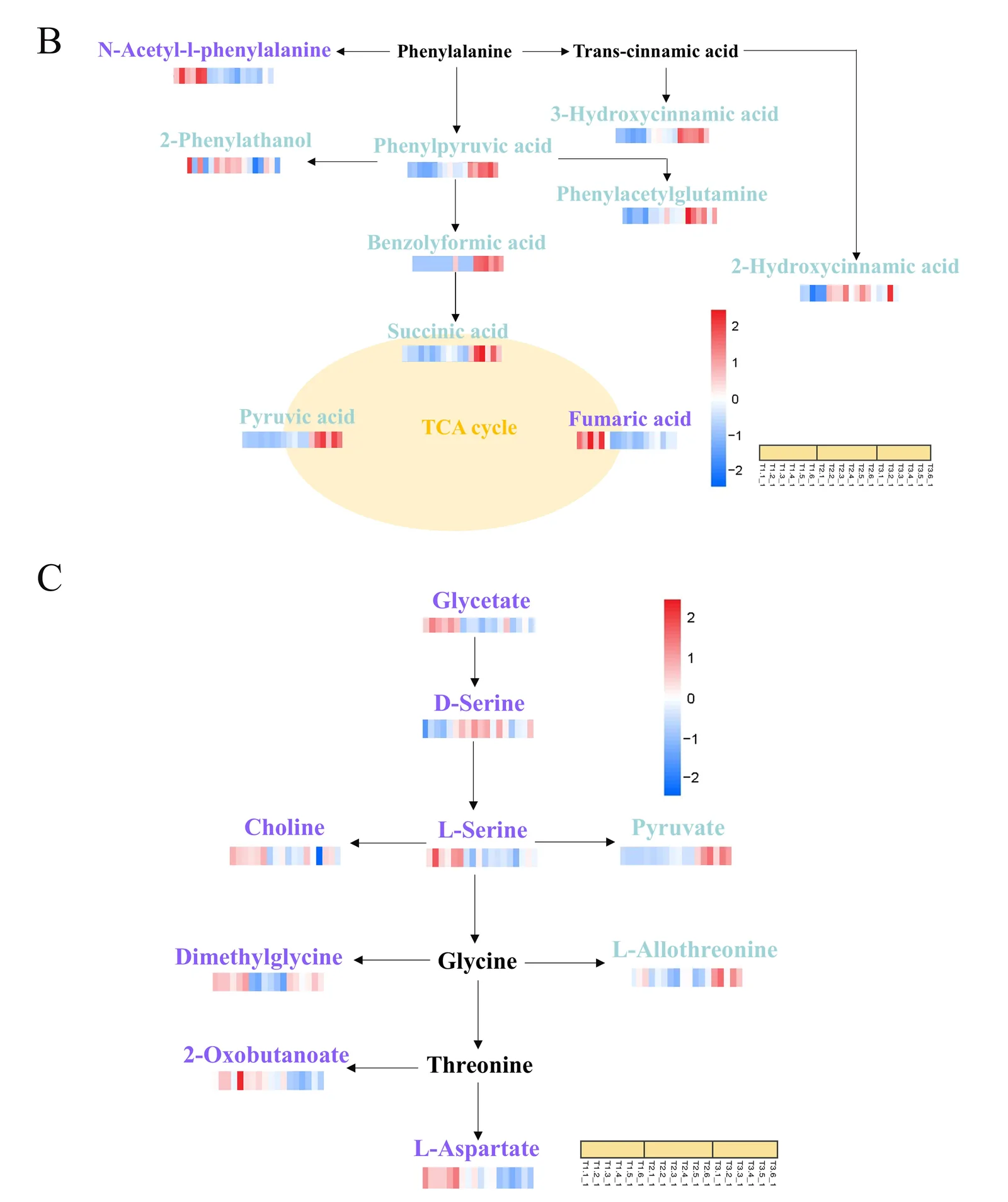
Three important metabolism pathways at different storage temperatures for saffron stigmas: (**A**) alanine, aspartate and glutamate metabolism; (**B**) phenylalanine metabolism; (**C**) glycine, serine and threonine metabolism (*n* = 6).

Consideration for alanine, aspartate and glutamate metabolism in different treatments was enriched. We conducted relative abundance analysis of differential metabolites in alanine, aspartate and glutamate metabolism (Figure 5A). We found that the relative abundance of important metabolites in alanine, aspartate and glutamate metabolism all showed an upregulated trend when saffron stigmas were stored in a higher-temperature environment (T1), and they were L-Aspartate, Asparagine, D-Aspartate, Fumarate, 4-Aminobutyric acid, and L-Glutaine, respectively. Previous studies have reported that amino acid metabolism is involved in secondary metabolite biosynthesis, signal transduction, stress response, pathogen defense, flavor formation, and other processes. Concurrently, amino acid metabolism functions as signaling molecules or participates in the regulation of plant signals to delay senescence in postharvest horticultural products [47]. It is noteworthy that arginine, glutamine, and asparagine are utilized for nitrogen storage and transport and are involved in the biosynthesis of polyamines such as spermidine, putrescine, and spermine, thereby contributing to plant responses to adverse stress. In this study, compared to T2 and T3, saffron stigmas stored in the T1 environment severely enhanced the relative abundance of different metabolites in arginine, glutamine, and asparagine metabolic pathways.

On the other hand, we found that phenylalanine metabolism was significantly enriched in saffron stigmas during postharvest storage (Figure 5B). Aromatic amino acids (such as phenylalanine) are the most extensively studied amino acids in postharvest storage research of fruits and vegetables. This may primarily be due to the high sensitivity of aromatic amino acid metabolism to various environmental stresses encountered by postharvest fruits and vegetables [47]. Previous studies reported phenylalanine metabolism serves as the precursor for the phenylpropanoid network, contributing to the biosynthesis of flavonoids and phenolic compounds to enhance antioxidant capacity and alleviate oxidative damage [46]. We could observe that these relative abundances of differential metabolites such as phenyl-pyruvic acid, 2-phenylathanol, benzolyformic acid, succinic acid, phenylacetylglutamine, 3-hydroxycinnamic acid, and pyruvic acid were increased in phenylalanine metabolism in T3 compared with T1 and T2. Therefore, combined with the crocin and picrocrocin contents of saffron stigmas, we speculated phenylalanine-derived metabolism exhibited antioxidant capacity and alleviated oxidative damage roles in saffron postharvest in T3.

On the other hand, the relative abundance of these differential metabolites, such as Glycetate, D-Serine, L-Serine, Choline, Dimethylglycine, 2-oxobutanoate, and L-aspartate in T1, was significantly increased, which had higher abundances compared with T2 and T3 (Figure 5C). Previous studies reported that serine played a primary role in responding to environmental stress, which maintained higher levels of serine and threonine to preserve the quality of postharvest horticultural products [47]. An increase in serine and threonine content in postharvest grapes, citrus fruits, and tomatoes helps to maintain antioxidant capacity and delays the senescence process of postharvest citrus and tomatoes [47,48,49,50]. Thereby, in this study, saffron stigmas stored in the T1 environment are associated with glycine, serine and threonine metabolism and result in producing more serine accumulation. Meanwhile, these differential metabolites in T1 vs. T2 were significantly enriched in glycine, serine and threonine metabolism compared with T2 vs. T3 and T1 vs. T3.

### 3.6. Important Medicinal Component Contents of Crocin and Picrocrocin in Saffron Stigmas Stored in Different Temperature Environments

Moratalla-López investigated dried saffron flower storage at 25 °C and 40 °C under 23%, 57%, and 75% relative humidity [51]. They found that high temperatures accelerated anthocyanin degradation in saffron petals, whereas flavonol remained more stable. These results are consistent with our study, confirming that saffron’s chemical components degrade more slowly in colder storage compared to higher-temperature conditions. Furthermore, Rahimi studied three storage conditions (room temperature with light, room temperature with dark, 4 °C in refrigerator) for stigmas storage and found that after 20 months, dark room-temperature storage performed best with higher crocin content, while humid conditions for storage performed worst [52]. However, currently, the optimal storage temperature for saffron has not yet been determined.

**Figure 6:**
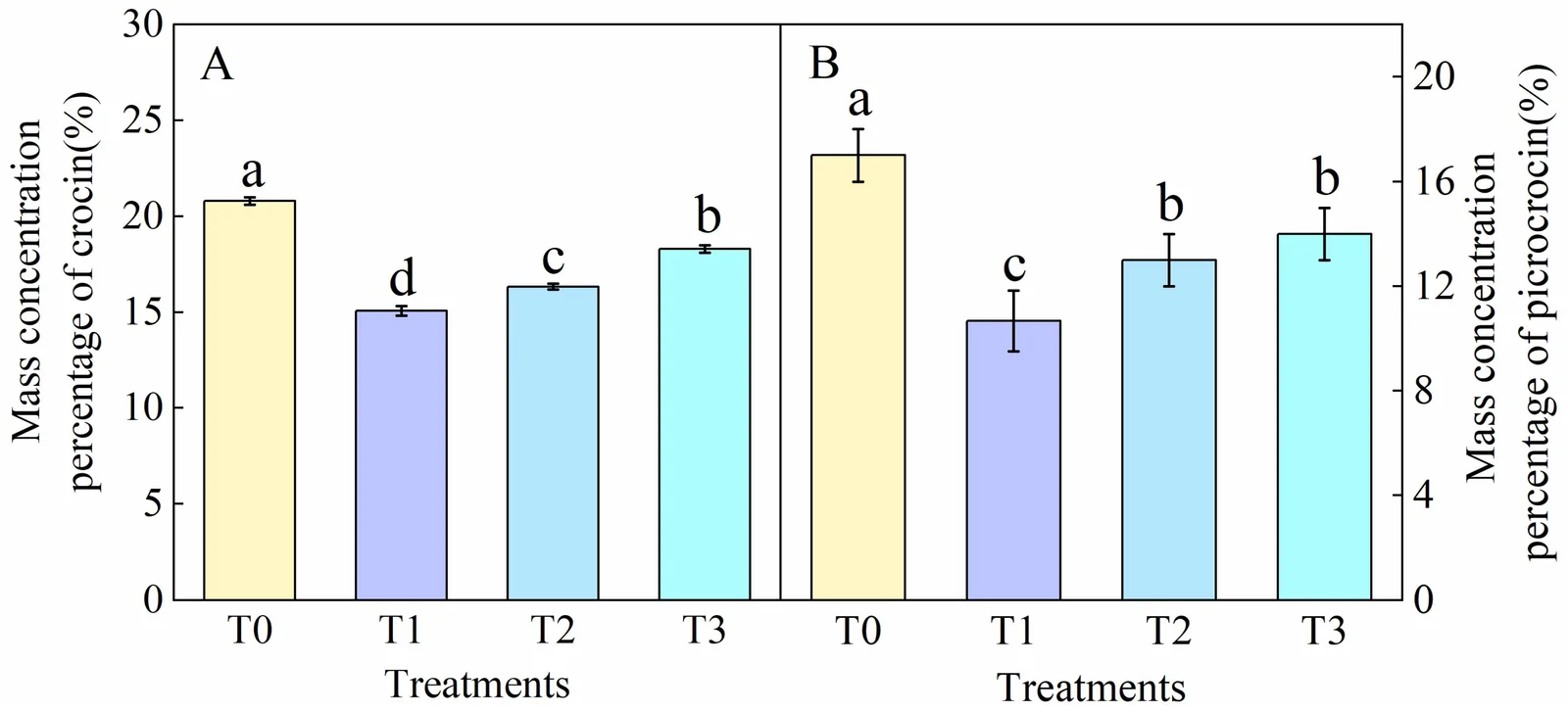
(**A**) mass concentration percentage of crocin; (**B**) mass concentration percentage of picrocrocin. Note: The mass concentration percentage of crocin and picrocrocin in saffron stigmas was determined immediately after drying and recorded as T0 without storage. Subsequently, dried stigmas of T0 were stored for one year under different storage temperatures (4 °C (T1), −20 °C (T2), and −80 °C (T3)), after which their mass concentration percentage of crocin and picrocrocin content was determined. All saffron stigma materials used for subsequent storage experiments were derived from the same batch of dried stigmas and subjected to identical drying procedures at the same time. Data are presented as mean ± SD. Statistical significance was determined by one-way analysis of variance (ANOVA) followed by Duncan’s multiple range test, and different lowercase letters indicate significant differences under different treatments (*p* < 0.05, *n* = 3).

In Figure 6, we further determine the mass concentration percentage of crocin and picrocrocin of stigmas under different stored environments. There was the highest mass concentration percentage of crocin and picrocrocin in T0, and they were 20.80% and 17%, respectively. After storage in different temperature environments for one year, it was found that the contents of mass concentration percentage of crocin and picrocrocin were significantly decreased along with storage environment temperature rises. They were decreased by 27.56%, 21.47%, and 12.01%, respectively, in T1, T2, and T3, compared with T0 in the mass concentration percentage of crocin. On the other hand, different temperatures in the storage environment had more effect on the function of picrocrocin content. In detail, the mass concentration percentage of picrocrocin in T1, T2, and T3 was significantly decreased by 37.25%, 23.52%, and 17.64%, respectively, compared with T0. In detail, there was a significantly higher content of mass concentration percentage of crocin and picrocrocin when stored in a −80 °C storage environment compared with in 4 °C and −20 °C. Our results indicate that a lower-temperature environment for saffron stigmas stored could efficiently avoid reducing important medicinal components, especially for the mass concentration percentage of crocin and picrocrocin. It is worth noting that there was no significant difference in the mass concentration percentage of picrocrocin between T2 and T3. If consumers lack a −80 °C storage environment to store saffron stigmas in their daily life, −20 °C is also an extremely suitable alternative.

On the other hand, among various secondary metabolites isolated from saffron, crocetin, crocin, picrocrocin and safranal represent the primary bioactive ingredients. Crocetin, a naturally occurring carotenoid dicarboxylic acid, is the precursor molecule of crocin, whereas picrocrocin, a monoterpene glycoside generated from zeaxanthin degradation, functions as the precursor of safranal. These metabolites endow saffron with its characteristic color, taste and fragrance, and simultaneously possess a range of health-beneficial bioactivities [44]. Saffron stigma is characterized by its water solubility and yellow coloration, conferred by crocin, and a bitter taste and a characteristic fragrance, which are caused by picrocrocin and safranal, respectively [53,54]. Our results indicate that lower temperatures can significantly prevent the reduction of medicinal components crocin and picrocrocin during long-term storage. Meanwhile, suitable lower temperatures such as −80 °C and −20 °C for stored stigmas could efficiently maintain the luster of stigmas and water solubility and yellow coloration when brewing.

### 3.7. Correlation Between Differential Metabolites and Key Quality Markers (Crocin, Picrocrocin)

**Figure 7:**
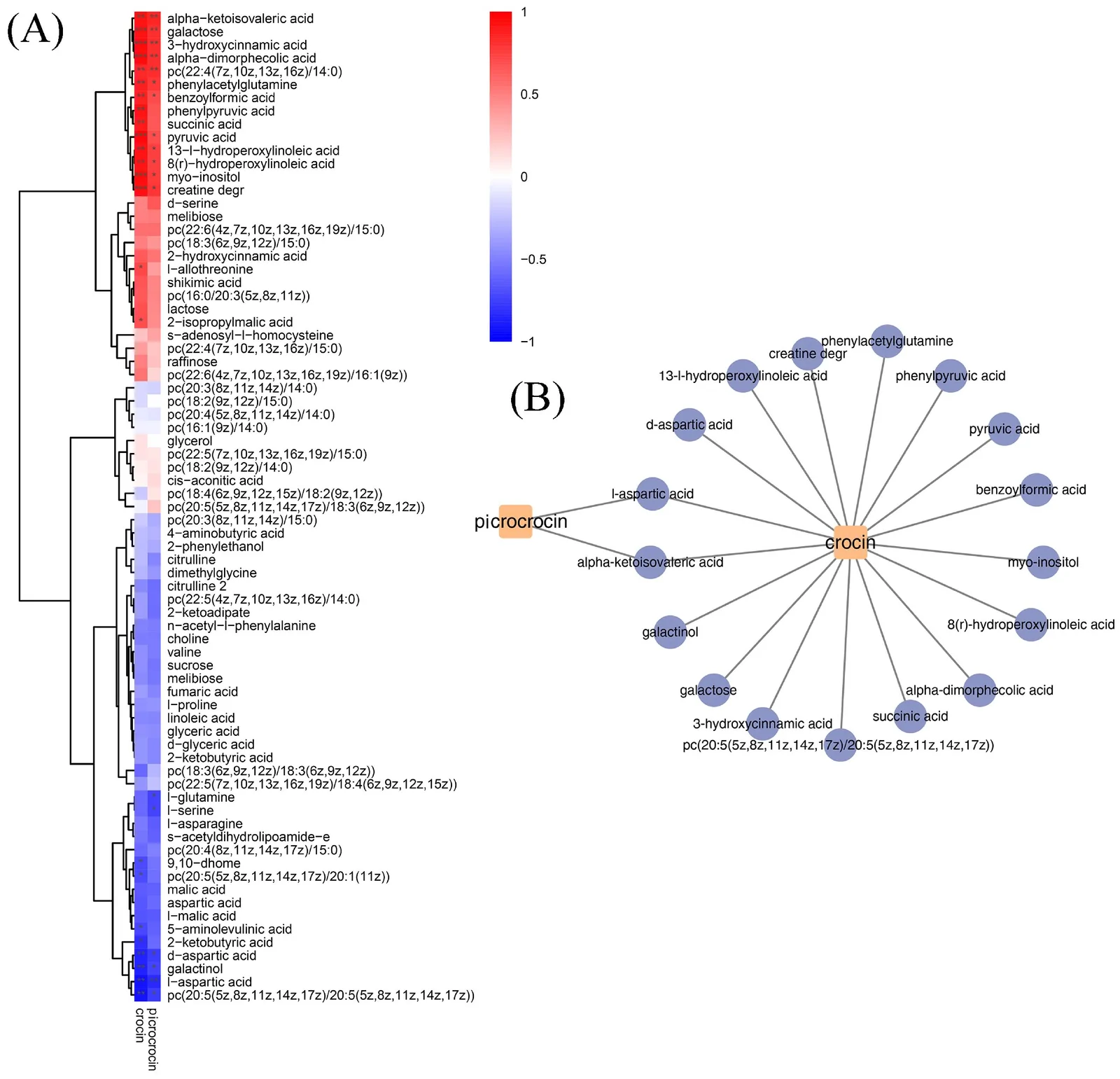
Correlation with mass concentration percentage of crocin and picrocrocin: (**A**) relevance clustering heatmap; (**B**) relevance network interaction analysis of stronger correlation of top 20 metabolites with mass concentration percentage of crocin and picrocrocin (|*r*| > 0.8, *p* < 0.05, *n* = 3). Note: To achieve one-to-one sample matching between metabolite profiles and quality parameters for correlation analysis, the metabolomic data of the two independent biological replicates were averaged, yielding three group-level datasets consistent with the three quality measurement samples.

It is worthy to notice that there were five common differential metabolism pathways between them, such as biosynthesis of amino acids, TCA cycle, glycine, serine and threonine metabolism, alanine, aspartate and glutamate metabolism, and phenylalanine metabolism. Meanwhile, linoleic acid metabolism and galactose metabolism also play important roles in the regulation of dried stigma storage, as shown in Figure 4. To further select out core differential metabolites of saffron stigmas in different temperature storage, we analyzed the correlation heatmap of these differential metabolites in seven metabolisms with mass concentration percentages of crocin and picrocrocin (Figure 7A). We found that there were about 75 differential metabolites that had a correlation with the mass concentration percentage of crocin and picrocrocin and 26 differential metabolites that had a significant correlation with two key quality markers (crocin and picrocrocin).

Thereby, we further conducted relevance network interaction analysis of the stronger correlation of the top 20 metabolites with the mass concentration percentage of crocin and picrocrocin in Figure 7B. Crocin is highly associated with six major categories of substances, including lipids, amino acids, TCA, carbohydrates, phenylpropanoids, and terpenoids, indicating a deep coupling between crocin accumulation and global carbon central metabolism as well as energy metabolism. Crocin exhibits a significant covariation with lipid peroxidation markers (linoleic acid hydroperoxide), indicating a strong association between Crocin accumulation and the cellular oxidative stress status, which may participate in scavenging reactive oxygen species and maintaining membrane system stability, such as 13-l-hydroperoxylinoleic acid and 8 (r)-hydroperoxylinoleic acid [55]. On the other hand, it is highly worthy to notice that crocin and picrocrocin all had strongly relevant network interactions with aspartic acid and alpha-ketoisovaleric acid, which played significantly important roles in saffron stigma quality when stored in different temperature conditions.

## 4. Conclusions

This study investigated different storage temperature conditions on stigma’s chemical substance activity after one year of storage analyzed by LC-MS and GC-TOF-MS. The results demonstrate that different storage temperatures can significantly influence the chemical activity of saffron stigmas, with lower storage temperatures being more effective in preserving their chemical component activity compared with higher temperatures. In detail, the top 15 substances had the most significant changes, such as capsiate, sarmentine, sesamolinol, nobiletin, prostaglandin F1α, vanillin, and α-ketoglutaric acid. Meanwhile, combined with KEGG enrichment analysis, amino acid metabolism is the most core responsive pathway throughout different T1, T2, and T3 temperature environments. Saffron stigmas were under stress in T1 compared with T2 and T3, which stimulated glycine, serine and threonine metabolism and arginine, glutamine, and asparagine metabolic pathways and resulted in producing more serine and regulating amino acid accumulation to enhance antioxidant ability and delay the senescence of postharvest dried stigmas. On the other hand, stigmas in T3 regulate phenylalanine metabolism and exhibit antioxidant capacity and alleviate oxidative damage roles in saffron postharvest, which maximize the retention of important medicinal substance components in saffron stigmas, such as crocin and picrocrocin. We selected two key differential metabolites namely aspartic acid and alpha-ketoisovaleric acid, which had significantly strong relevance network interaction with key quality markers (crocin, picrocrocin) of saffron stigmas. Meanwhile, the highest contents of mass concentration percentage of crocin and picrocrocin were found in T3. Compared to freshly dried saffron stigmas, storing saffron stigmas at −80 °C significantly resulted in a reduction of only 12% and 17% in crocin and picrocrocin content, respectively. Therefore, we recommend that a lower-temperature environment (−80 °C) should be selected for saffron stigmas storage if they need to be stored for a long time for ensuring medicinal efficacy. Furthermore, from a consumer perspective, a −20 °C environment is also a favorable option for the storage of saffron stigmas in their daily life. Our research provides technical guidance and a theoretical basis for the long-term storage of saffron stigmas for both consumers and producers.

## Data Availability

The original contributions presented in this study are included in the article/Supplementary Materials. Further inquiries can be directed to the corresponding authors.

## Supplementary Materials

The following supporting information can be downloaded at: https://www.mdpi.com/article/10.3390/foods15203758/s1, Figure S1: OPLS-DA analysis; Figure S2: K-means analysis with nine clusters.
