# Supplementary material for: The Effects of Different Storage Temperatures on Chemical Substance Changes in Saffron Stigmas Analyzed by LC-MS and GC-TOF-MS
Source: Foods. 2026 Oct 21;15(20):3758. doi: 10.3390/foods15203758 (PMC13704096; doi:10.3390/foods15203758)
Supplement: Supplementary file 1 [file foods-15-03758-s001.zip › foods-4580005-supplementary.pdf]

Figure Supplementary

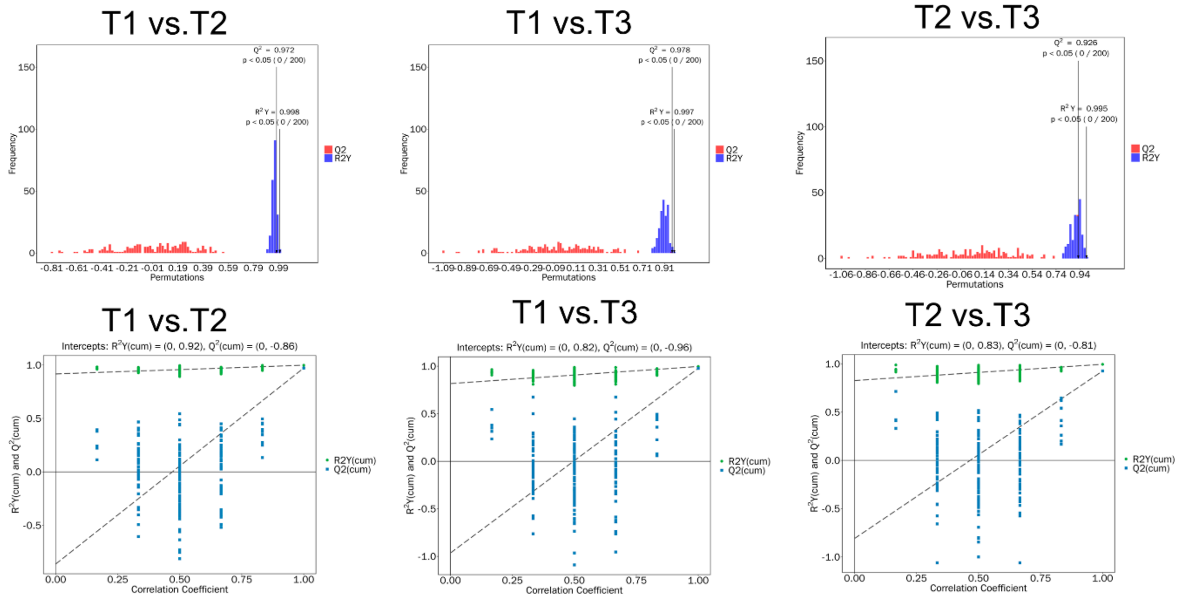

Figure S1. OPLS-DA Analysis

Note: Cross-validation Design: A 7-fold cross-validation is employed, and the Q^2 value after cross-validation is reported. Permutation Test: 200 permutation tests are conducted, and the permutation P-values for R^2Y and Q^2 are reported, along with the permutation test plot.

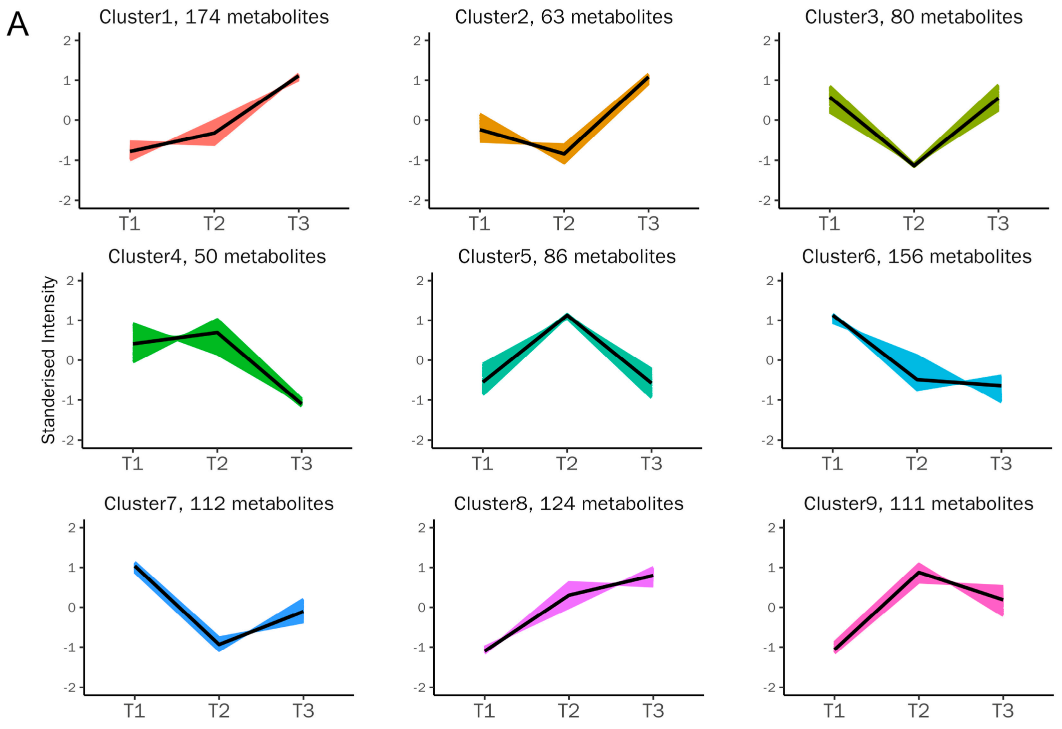

Figure S2. K-means analysis with nine clusters
